# Supplementary material for: Switching and Combining Device-Aided Therapies in Advanced Parkinson’s Disease: A Double Centre Retrospective Study
Source: Brain Sci. 2022 Mar 2;12(3):343. doi: 10.3390/brainsci12030343 (PMC8946829; doi:10.3390/brainsci12030343)
Supplement: Supplementary file 1 [file brainsci-12-00343-s001.zip › Supplementary Tables 2-4.pdf]

Supplementary Table S2. Gender differences in patients that switched and combined Device Aided Therapy (DAT). The median values and range (in brackets) are presented. PD=Parkinson's disease, GI= Global Improvement.

	Age at PD onset	PD duration at 1st DAT	Age at 1st DAT	Duration of 1st DAT	Age at 2nd DAT	Duration 2nd DAT	GI after 1st DAT	GI after 2nd DAT
Females (n=12)	48.5 (29-69)	11.5 (3-20)	63 (38-72)	1.75 (0.08-11)	66.5 (44-76)	3 (0.08-8)	1.5 (1-5)	2 (1-4)
Males (n=18)	50.5 (36-66)	10 (6-28)	60 (44-75)	3 (0.5-18)	67 (39-79)	2.5 (0.08-9)	2 (1-6)	2 (1-4)
Mann-Whitney U	21.5	18.5	23	17.5	18.5	19	23.5	15.5
p-value	0.746	0.474	0.897	0.398	0.473	0.511	0.945	0.249

Supplementary Table S3. Differences between between different groups of patients that switched from one to another Device Aided Therapy (DAT). The median values and range (in brackets) are presented. **Significant values are written in bold.** PD=Parkinson's disease, GI= Global Improvement, CSAI=Continuous subcutaneous apomorphine infusion, LCIG=Levodopa-carbidopa intestinal gel infusion, and STN-DBS=Deep brain stimulation of the subthalamic nucleus.

	Age at PD onset	PD duration at 1st DAT	Age at 1st DAT	Duration of 1st DAT	Age at 2nd DAT	Duration of 2nd DAT	GI after 1st DAT	GI after 2nd DAT	Wicloxon *Z* *p*-value
LCIG-to-STN-DBS (n=7)	45 (29-50)	11 (9-28)	59 (38-69)	4 (1-11)	66 (44-73)	3 (2-8)	3 (1-3)	2 (1-3)	-0.55, p=0.581
CSAI-to-STN-DBS (n=5)	43 (36-52)	8 (8-13)	52 (44-65)	1 (1-2)	54 (39-67)	2 (1-9)	2 (1-2)	1 (1-3)	-1.00, p=0.317
CSAI-to-LCIG (n=8)	55 (48-66)	12 (7-20)	70 (58-74)	1.5 (0.5-5)	72 (59-77)	2.75 (0.5-5)	2 (1-3)	2 (1-3)	0.00, p=1.000
Kruskal Wallis *H*	**9.03**	3.14	**8.25**	3.26	**7.66**	0.83	0.70	2.35	/
p-value	**0.011**	0.110	**0.016**	0.196	**0.022**	0.662	0.705	0.309	/

Supplementary Table S4. Differences between patients who switched Device Aided Therapy (DAT) and patients who combined DAT. The median values and range (in brackets) are presented. **Significant values are written in bold.** PD=Parkinson's disease, GI= Global Improvement.

	Age at PD onset	PD duration at 1st DAT	Age at 1st DAT	Duration of 1st DAT	Age at 2nd DAT	Duration of 2nd DAT	GI after 1st DAT	GI after 2nd DAT	Wilcoxon *Z* *p*-value
Switched (n=24)	**49 (29-66)**	**10 (7-28)**	60 (38-74)	**1.75 (0.08-18)**	66 (39-77)	3 (0.08-9)	**2(1-6)**	2(1-4)	-0.91, p=0.327
Combined (n=6)	**55 (46-69)**	**7 (3-11)**	62 (57-75)	**5 (3-10)**	68 (64-79)	3 (0.08-5)	**1 (1-1)**	1.5 (1-2)	-1.72, p=0.083
Mann-Whitney *U*	**30.5**	**29.0**	57.5	**27.5**	41.5	67.5	**18.0**	49.5	/
p-value	**0.031**	**0.025**	0.452	**0.020**	0.113	0.813	**0.003**	0.251	/

.
